# Interleukin-1 and TRAF6-dependent activation of TAK1 in the absence of TAB2 and TAB3

**DOI:** 10.1042/BCJ20170288

**Published:** 2017-06-26

**Authors:** Jiazhen Zhang, Thomas Macartney, Mark Peggie, Philip Cohen

**Affiliations:** MRC Protein Phosphorylation and Ubiquitylation Unit, School of Life Sciences, University of Dundee, Dundee DD1 5EH, U.K.

**Keywords:** interleukin-1, TAB1, TAB2, TAK1, TRAF6, ubiquitin

## Abstract

Interleukin-1 (IL-1) signaling induces the formation of Lys63-linked ubiquitin (K63-Ub) chains, which are thought to activate the ‘master’ protein kinase TGFβ-activated kinase 1 (TAK1) by interacting with its TAK1-binding 2 (TAB2) and TAB3 subunits. Here, we report that IL-1β can also activate the TAB1–TAK1 heterodimer present in TAB2/TAB3 double knockout (DKO) IL-1 receptor-expressing cells. The IL-1β-dependent activation of the TAB1–TAK1 heterodimer in TAB2/3 DKO cells is required for the expression and E3 ligase activity of tumor necrosis factor receptor-associated factor 6 (TRAF6) and is reduced by the small interfering RNA (siRNA) knockdown of ubiquitin conjugating 13 (Ubc13), an E2-conjugating enzyme that directs the formation of K63-Ub chains. IL-1β signaling was restored to TAB1/2/3 triple KO cells by the re-expression of either TAB1 or TAB2, but not by an ubiquitin binding-defective mutant of TAB2. We conclude that IL-1β can induce the activation of TAK1 in two ways, only one of which requires the binding of K63-Ub chains to TAB2/3. The early IL-1β-stimulated, TAK1-dependent activation of p38α mitogen-activated protein (MAP) kinase and the canonical IκB kinase (IKK) complex, as well as the NF-κB-dependent transcription of immediate early genes, was similar in TAB2/3 DKO cells and TAB2/3-expressing cells. However, in contrast with TAB2/3-expressing cells, IL-1β signaling was transient in TAB2/3 DKO cells, and the activation of c-Jun N-terminal kinase 1 (JNK1), JNK2 and p38γ was greatly reduced at all times. These observations indicate a role for TAB2/3 in directing the TAK1-dependent activation of MAP kinase kinases that switch on JNK1/2 and p38γ MAP kinases. These observations and the transient activation of the TAB1–TAK1 heterodimer may explain why IL-1β-dependent IL-8 mRNA formation was abolished in TAB2/3 DKO cells.

## Introduction

Interleukin-1 (IL-1) has a central role in regulating immune and inflammatory responses to infection and tissue damage [1]. IL-1 signaling is initiated by the recruitment of MyD88 (Myeloid Differentiation primary response gene 88) to the IL-1 receptor (IL-1R) complex, which is followed by the interaction of IL-1R-associated kinase 4 (IRAK4) with MyD88 and IRAK1 and/or IRAK2 with IRAK4, to form an oligomeric complex termed the Myddosome [2,3].

IRAK1 activates several E3 ubiquitin ligases. First, it interacts with tumor necrosis factor receptor-associated factor 6 (TRAF6) [4] and induces its dimerization and activation [5]. Second, it phosphorylates Pellino1 and Pellino2, converting them from inactive into active E3 ligases [6–8]. TRAF6 and Pellino1/2 can then form productive complexes with the E2-conjugating enzyme complex termed ubiquitin conjugating 13 (Ubc13)-Uev1a to generate Lys63-linked ubiquitin (K63-Ub) chains [9]. In contrast, the pseudokinase IRAK2, which lacks kinase catalytic activity, can activate TRAF6 [10], but would not be expected to be capable of activating Pellino1 and Pellino2 [9].

It is widely accepted that IL-1-generated K63-Ub chains activate TGFβ-activated kinase 1 (TAK1, also called MAP3K7) [11,12]. Cells express two TAK1 complexes, which comprise the TAK1 catalytic subunit (TAK1), TAK1-binding protein 1 (TAB1) and either TAB2 or TAB3 [13]. The K63-Ub chains interact specifically with the C-terminal Npl4 Zinc Finger (NZF) domain present in TAB2 and TAB3 [14,15] triggering the activation of TAK1 complexes *in vitro* [11,12].

One role of TAK1 is to activate mitogen-activated protein (MAP) kinase (MAPK) kinase 4 (MKK4) and MKK7 (also called MAP2K4 and MAP2K7) [16,17], which activate c-Jun N-terminal kinase 1 (JNK1) and JNK2, while another is to initiate the activation of IKKβ [18–20]. The dual phosphorylation of IKKβ at Ser177 and Ser181 permits IKKβ to phosphorylate and activate the transcription factors nuclear factor kappa B (NF-κB) [21] and interferon regulatory factor 5 (IRF5) [22,23] and the protein kinase tumor progression locus 2 (Tpl2, also called MAP3K8). The IKKβ-dependent activation of Tpl2 requires the phosphorylation of its p105/NF-κB1 subunit [24,25] and Tpl2 itself [26]. Tpl2 activates MEK1 (mitogen-activated kinase kinase or ERK kinase), MEK2 (also called MAP2K1 and MAP2K2, respectively), MKK3 and MKK6 (also called MAP2K3 and MAP2K6, respectively) [27,28]. MEK1 and MEK2 activate extracellular signal-regulated kinase 1 (ERK1) and ERK2, whereas MKK3 and MKK6 appear to operate redundantly with MKK4 to activate p38α MAP kinase. The relative importance of MKK3, MKK4 and MKK6 in activating p38α MAP kinase varies with cell type and cell stimulus [28].

If the interaction of K63-Ub chains with TAB2 and TAB3 is required to activate the heterotrimeric TAB1–TAK1–TAB2 and TAB1–TAK1–TAB3 complexes, then the activation of TAK1 should not occur in cells devoid of TAB2 and TAB3 expression. Here, we have made extensive use of CRISPR (clustered regularly interspaced short palindromic repeat)/Cas9 (CRISPR-associated protein 9) gene-editing technology to examine this hypothesis in IL-1R* cells, a human embryonic kidney (HEK) 293 cell line that stably expresses low levels of IL-1R. IL-1β signaling in these cells is dependent on the expression of IRAK1 and requires both the expression and protein kinase activity of TAK1 [9]. We report that IL-1 activates the TAB1–TAK1 heterodimer rapidly and robustly in IL-1R* cells lacking expression of TAB2 and TAB3, but activation is more transient than in TAB2/3-expressing cells. These and other findings demonstrate that IL-1 activates TAK1 in two ways, only one of which requires the interaction of K63-Ub chains with TAB2/3.

## Materials and methods

### DNA constructs and proteins

Recombinant DNA procedures, restriction digests and ligations were performed using standard protocols. All PCRs were carried out using KOD Hot Start DNA polymerase (Merck Millipore). DNA sequencing was performed by the DNA Sequencing Service, School of Life Sciences, University of Dundee (www.dnaseq.co.uk). All clones were human unless stated otherwise.

DNA clones encoding TAB1 (DU51103), TAB2 (DU46500), TAB2[T674A/F675A] (DU46511), TRAF6 (DU51583), TRAF6[C70A] (DU51585) and TRAF6[L74H] (DU51584) were inserted into pRetroX-Tight-Puromycin vectors [20]. DNA clones encoding IL-1R1 (DU46481), FLAG-TRAF6 (DU32495), FLAG-TRAF6[L74H] (DU46743) and FLAG-TRAF6[120-522] lacking the Really Interesting New Gene (RING) domain (DU51445) were inserted into a pBABE vector [20].

Human IL-1β (DU8685) [29] was expressed in *Escherichia coli* and purified by the Protein Production Team of the Medical Research Council Protein Phosphorylation and Ubiquitylation Unit (MRC-PPU). The DNA clones and proteins generated for the present study have been assigned [DU] numbers and can be ordered from the reagents section of the MRC-PPU website (https://mrcppureagents.dundee.ac.uk/).

### Antibodies

Polyclonal antibodies against TAK1 (S828A, first bleed) and TAB1 (S823A, first bleed) were raised in sheep by the antibody production team of the MRC-PPU. An antibody that recognizes IKKα phosphorylated at Ser176 and Ser180, and IKKβ phosphorylated at Ser177 and Ser181 (#2697), an antibody recognizing p38α phosphorylated at Thr180 and Tyr182 and p38γ MAP kinase phosphorylated at Thr183 and Tyr185 (#9211), and antibodies recognizing TAK1 phosphorylated at Thr187 (#4536), p105/NF-κB1 phosphorylated at Ser933 (#4806), ERK1 and ERK2 phosphorylated at Thr202/Tyr204 (#9101), and antibodies recognizing all forms of TAK1 (#4505), TAB2 (#3745), TAB3 (#14241), p38α MAP kinase (#9212), X-linked inhibitor of apoptosis protein (XIAP; #2012), cellular inhibitor of apoptosis protein (cIAP1; #7056) and glyceraldehyde 3-phosphatase dehydrogenase (GAPDH; #2118) were purchased from Cell Signaling Technology. An antibody recognizing TAB1 (#ab151408) was obtained from Abcam, an antibody recognizing Ubc13 (#37-1100) and a phospho-specific antibody recognizing JNK1 and JNK2 phosphorylated at Thr183 and Tyr185 (#44682) were from Invitrogen, an antibody recognizing IKKβ (#05-535) was from Merck Millipore and an antibody recognizing TRAF6 (#sc-7221) was from Santa Cruz Biotechnology. A phospho-specific antibody that recognizes IRAK4 phosphorylated at Thr345 and Ser346 [30] was a generous gift from Dr Vikram Rao, Pfizer, Boston, U.S.A. Secondary antibodies coupled to horseradish peroxidase were obtained from Thermo Scientific.

### Generation of IL-1R* cells lacking expression of TRAF6 and/or TAB subunits using the CRISPR/Cas9 gene-editing technology

IL-1R* cells expressing low levels of the IL-1R were generated as described recently [9]. The guide (g) RNAs used to target the genes encoding TRAF6, TAB1, TAB2, TAB3 and XIAP1 are listed in Supplementary Figure S1. The gRNA plasmids targeting each gene were pooled and 10 µg was used to transfect IL-1R* cells for 8 h using the GeneJuice transfection reagent (Merck Millipore). Doxycycline was then added to the cells to a final concentration of 1.0 µg/ml, and a further 18 h later, the cells were again transfected with the same amounts of gRNA plasmids. After 48 h, cells were single-cell plated into 96-well plates and left until colonies began to form (2–3 weeks). The loss of expression was analyzed by immunoblotting after their immunoprecipitation (TAB1 and TAB3). Multiple TAB1 knockout (KO) and TAB3 KO clones were generated, a few of which were selected for further study. Double KO (DKO) IL-1R* cells lacking expression of both TAB2 and TAB3 were generated by targeting TAB3 KO IL-1R* cells with a pair of gRNAs specific for TAB2. Triple knockout (TKO) IL-1R* cells lacking any expression of TAB1, TAB2 and TAB3 were generated by targeting the TAB2/TAB3 double KO cells with a pair of gRNAs for TAB1. DKO cells lacking expression of TRAF6 and TAB1 and TKO cells lacking expression of TRAF6, TAB2 and TAB3 were generated by targeting TAB1 KO cells or TAB2/TAB3 DKO cells with a pair of gRNAs for TRAF6. TKO cells lacking expression of TAB2, TAB3 and XIAP were generated by targeting TAB2/3 DKO cells with a pair of gRNAs for XIAP. The DKO or TKO cells were produced by CRISPR/Cas9 technology using an improved procedure. One pair of gRNAs was generated to target TAB1, TAB2, TRAF6 or XIAP. The antisense gRNA was introduced to the vector encoding the Cas9[D10A] mutant, which only cleaves one strand of the DNA molecule complementary to the gRNA. In contrast, the sense gRNA was inserted into a plasmid containing a puromycin-resistance gene. Each gRNA plasmid (1.0 μg) was mixed with 1.0 ml of serum-free DMEM and 0.02 ml of polyethylenimine (1.0 mg/ml), and after incubation for 20 min at 20°C, the solution was added to the cells dropwise for transfection. After 48 h, the medium was replaced with fresh medium containing 2.0 μg/ml puromycin. The cells were then single-cell plated into 96-well plates and left until colonies began to form (2–3 weeks). The mutational efficiency was analyzed by immunoblotting of the cell extracts for the relevant proteins.

### Re-expression of components of the TAK1 complex and TRAF6

TAB1/2/3 TKO cells expressing HA-tagged versions of TAB1, TAB2, the TAB2[T674A/F675A] mutant, TRAF6 and the E3 ligase-inactive TRAF6 mutants, TRAF6[C70A] and TRAF6[L74H], and TRAF6[120-522] lacking the N-terminal 119 amino acid residues containing the RING domain were generated by retroviral transduction [20]. Viruses encoding the gene of interest and the Tet-On protein were harvested 48 h after transfection, diluted four-fold with fresh medium and incubated with the cells for 24 h in the presence of 2.0 μg/ml protamine sulfate (Sigma). Fresh medium containing 1 mg/ml G418 (Tet-On) and 2.0 μg/ml puromycin (gene of interest) was added to select the transduced cells. To induce gene expression, cells were cultured for 16 h in the presence of 0.1–1.0 µg/ml doxycycline.

### Quantitative RT-PCR

IL-1R* cells were seeded into 24-well plates at a final concentration of 1.5 × 10^5^ per ml, and the RNA was extracted using an RNA MicroElute kit from VWR (R6831-01). RNA was reverse-transcribed using the iScript cDNA synthesis kit from Bio-Rad (170-8891). Quantitative PCR was performed using SsoFast EvaGreen Supermix from Bio-Rad (172-5204) in the CFX384 (Bio-Rad). Primer sequences:

IL-8 forward: 5′-ATAAAGACATACTCCAAACCTTTCCAC-3′;

IL-8 reverse: 5′-AAGCTTTACAATAATTTCTGTGTTGGC-3′;

IκBα forward: 5′-GATCCGCCAGGTGAAGGG-3′;

IκBα reverse: 5′-GCAATTTCTGGCTGGTTGG-3′;

A20 forward: 5′-GCAGAAAAGCCGGCTGCGTG-3′;

A20 reverse: 5′-CGCTGGCTCGATCTCAGTTGCT-3′.

Normalization was performed using 18S RNA and the ΔΔ*C*_t_ method. Primer sequences used were 18S forward: 5′-GTAACCCGTTGAACCCCATT-3′; 18S reverse: 5′-CCATCCAATCGGTAGTAG-CG-3′.

### Other materials and methods

The IAP inhibitor GT12911 (also called compound A) [31–33] was provided by Dr Stephen Condon (Tetralogic Pharmaceutical Corp, Malvern, PA, U.S.A.). The TAK1 inhibitor NG25 was provided by Dr Nathanael Gray (Dana Farber Cancer Institute, Boston, U.S.A.). Cell culture, cell stimulation, IL-8 secretion, cell lysis, SDS–PAGE and immunoblotting [9] and siRNA knockdown of Ubc13 [29] were performed as described.

### Reproducibility and statistical analysis

All experiments in the present paper were repeated at least three times with similar results, unless stated otherwise. Statistical analyses were performed with the GraphPad Prism Software, and quantitative data in graphs and bar charts are presented as the arithmetic mean ± SEM. Statistical significance of differences between experimental groups was assessed in all graphs and bar charts, using the one-way ANOVA, unless indicated otherwise. Differences were considered significant if *P* < 0.05; *P < 0.05, **P < 0.01, ***P < 0.001, n.s., not statistically significant.

## Results

### IL-1 activates the TAB1–TAK1 heterodimer in TAB2/3 DKO cells

**Figure 1.**
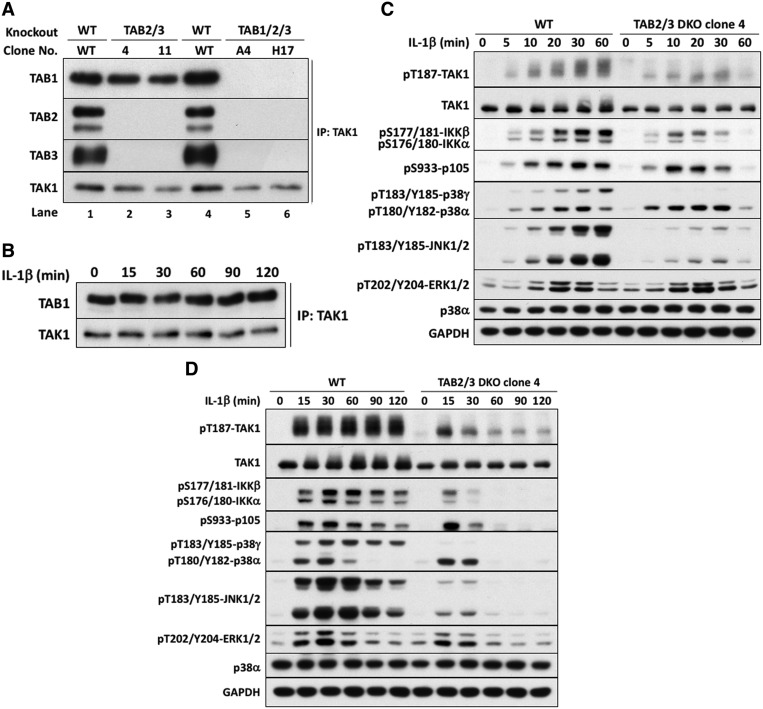
IL-1β signaling in TAB2/3 DKO cells. (**A**) Generation of IL-1R* cells lacking TAB2 and TAB3 or all three TAB subunits. TAK1 was immunoprecipitated from the extracts of WT IL-1R* cells (lane 1), two different clones (4 and 11) of cells devoid of TAB2 and TAB3 (lanes 2 and 3) and two different clones (A4 and H17) lacking expression of all three TAB components. Immunoprecipitates were subjected to SDS–PAGE, and immunoblotting as in the Methods with antibodies recognizing TAK1 or each TAB protein. (**B**) TAB2/3 DKO cells (clone 4 from **A**) were stimulated with 5 ng/ml IL-1β for the times indicated, and TAK1 immunoprecipitated from the extracts and processed as in **A**. (**C** and **D**) WT or TAB2/3 DKO IL-1R* cells (clone 4 from **A**) were stimulated with IL-1β for up to 1 h (**C**) or 2 h (**D**) as in **B**, and cell extracts analyzed by SDS–PAGE and immunoblotting with phospho-specific antibodies recognizing phosphorylated (p) serine (pS), threonine (pT) and tyrosine (pY) residues in the activation loops of TAK1, IKKα/β, p105/NF-κB1, JNK1/2, p38α/p38γ and ERK1/ERK2 MAP kinases, or with antibodies recognizing all forms of TAK1, p38α and GAPDH.

To investigate whether the interaction of K63-Ub chains with TAB2 and TAB3 was required to activate TAK1, we generated IL-1R* cells lacking any expression of TAB2 and TAB3, hereafter termed TAB2/3 ‘DKO’ cells (Figure 1A, **lanes 2 and 3**). In these cells, TAB1 remained associated with TAK1 even after stimulation for as long as 2 h with IL-1β (Figure 1B).

The activation of TAK1 requires its phosphorylation at the activation loop residue Thr187 [34]. Surprisingly, IL-1β induced the phosphorylation of TAK1 at Thr187 in TAB2/3 DKO cells and, consequently, also stimulated the phosphorylation of the canonical IKK complex and its substrate p105/NF-κB1, as well as p38α MAP kinase. The degree of activation of the IKK complex and p38α MAP kinase in TAB2/3 DKO and wild-type (WT) IL-1R* cells was similar for up to 20 min after IL-1β stimulation (Figure 1C). Thus, IL-1β can activate the TAB1–TAK1 heterodimer in DKO cells by a mechanism that is independent of the binding of K63-Ub chains to TAB2 and TAB3.

Interestingly, the IL-1β-dependent phosphorylation of JNK1, JNK2 and p38γ MAP kinase was greatly reduced in the TAB2/3 DKO cells compared with TAB2/3-expressing IL-1R* cells (Figure 1C). JNK1 and JNK2 are activated by MKK4 and MKK7 (see Introduction), suggesting a potential role for TAB2/3 in directing TAK1 complexes to one or both of these substrates. Which MKK family member activates p38γ MAP kinase in these cells is unknown.

**Figure 2.**
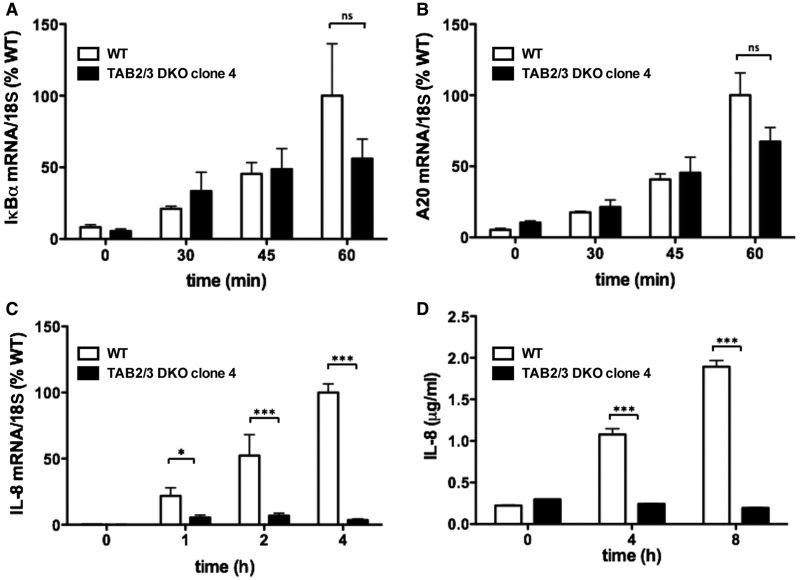
IL-1β-dependent gene transcription in TAB2/3 DKO IL-1R* cells. (**A**–**C**) Cells were stimulated with IL-1β as in Figure 1, and at the times indicated, the mRNA encoding IκBα (**A**), A20 (**B**) and IL-8 (**C**) was measured by qRT-PCR relative to 18S ribosomal mRNA and normalized to the level found in WT cells stimulated with IL-1β. The results are presented as arithmetic mean ± SEM for three independent experiments, each performed in triplicate. (**D**) As in **C**, except that IL-8 secreted into the culture medium was measured by ELISA.

The IL-1β-dependent phosphorylation of TAK1, IKKα/β and p38α MAP kinase in the TAB2/3 DKO cells was transient and had almost returned to basal levels after 60 min, but the IL-1β-dependent activation of ERK1 and ERK2, which is controlled by the protein kinase Tpl2 (see Introduction), was unaffected. In contrast, IL-1β signaling was sustained for at least 2 h in TAB2/TAB3-expressing IL-1R* cells (Figure 1D). Similar results were obtained with a second clone of TAB2/3 DKO cells that was isolated independently (Supplementary Figure S2). Consistent with these findings, the IL-1β-stimulated transcription of two NF-κB-dependent immediate early genes, IκBα (inhibitor of kappa B alpha; Figure 2A) and A20 (Figure 2B), was similar in TAB2/3 DKO cells and TAB2/3-expressing IL-1R* cells for up to 45 min. In contrast, the production of IL-8 mRNA (Figure 2C) and IL-8 secretion (Figure 2D) was reduced drastically in the TAB2/3 DKO cells. This may be explained by the transient activation of IKKα/IKKβ and/or p38α MAP kinase, and/or by the weak activation of JNK1/2 and p38γ MAP kinase in these cells.

The IL-1-stimulated phosphorylation of TAK1 at Thr187 in the TAB2/3 DKO cells was not accompanied by a decreased electrophoretic mobility of TAK1 during SDS–PAGE, in contrast with the TAB2/3-expressing IL-1R* cells (Figure 1C,D), suggesting that there are amino acid residues in TAK1, distinct from Thr187, that are phosphorylated in TAB2/3-expressing but not in TAB2/3 DKO cells.

### The IL-1-dependent activation of the TAB1–TAK1 heterodimer requires TRAF6 and the formation of ubiquitin chains

**Figure 3.**
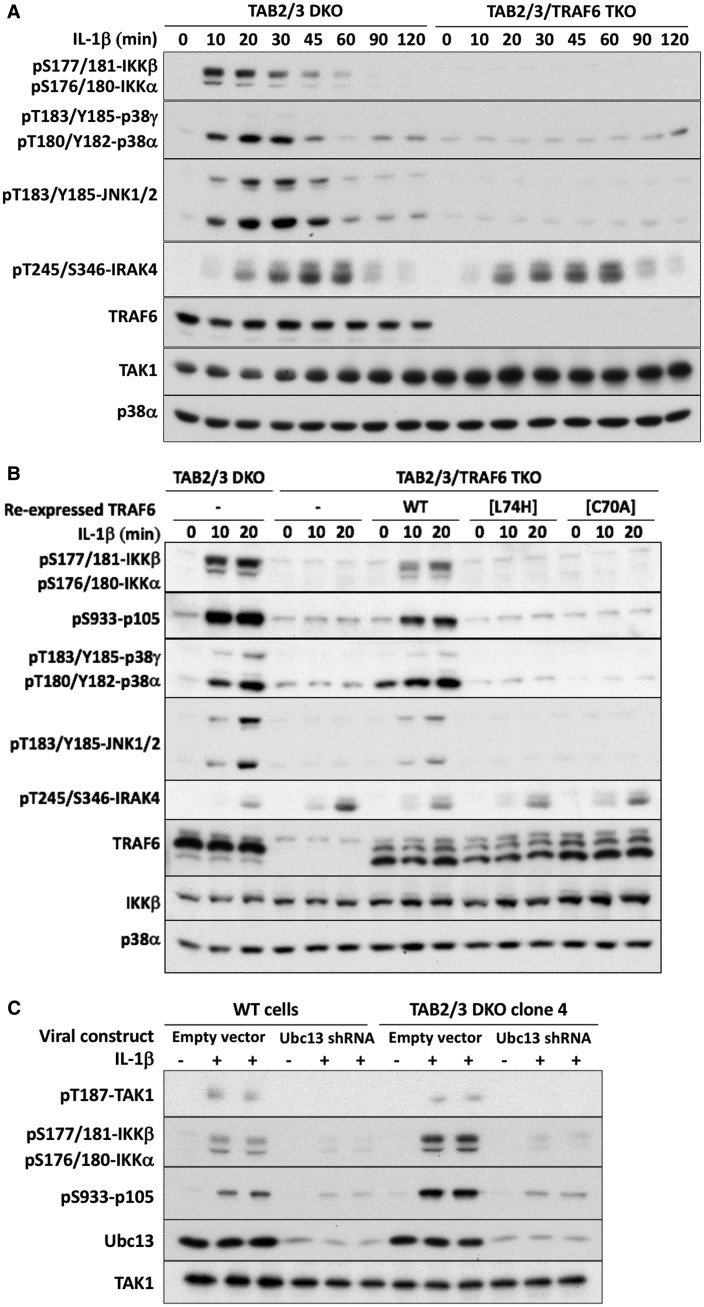
TRAF6 E3 ligase activity is required for the IL-1β-dependent activation of the TAB1–TAK1 heterodimer in TAB2/3 DKO cells. (**A**) TAB2/3 DKO (clone 4 from Figure 1A) and TAB2/TAB3/TRAF6 TKO IL-1R* cells were stimulated with IL-1β for the times indicated, and cell extracts subjected to SDS–PAGE and immunoblotting with the antibodies used in Figure 1 and an antibody recognizing IRAK4 phosphorylated at Thr245 and Ser346. (**B**) As in **A**, except that WT TRAF6 or the indicated E3-ligase-inactive mutants of TRAF6 were re-expressed in the TAB2/TAB3/TRAF6 TKO IL-1R* cells. (**C**) WT and TAB2/3 DKO cells were incubated for 72 h with siRNA against Ubc13 and stimulated for 10 min with 5 ng/ml IL-1β. Other details are as in **A**.

To investigate how the TAB1–TAK1 heterodimer might be activated, we ablated the expression of TRAF6 in the TAB2/3 DKO cells. IL-1β-dependent signaling ‘downstream’ from TRAF6 was abolished in these TAB2/TAB3/TRAF6 triple KO cells, but signaling ‘upstream’ of TRAF6 was not affected, as shown by the unimpaired phosphorylation of IRAK4 (Figure 3A). These experiments establish that the expression of TRAF6 is essential for the IL-1β-dependent activation of the TAB1–TAK1 heterodimer. Previously, we reported that E3 ligase-inactive mutants of TRAF6 partially restore IL-1 signaling to TRAF6 KO TAB2/3-expressing IL-1R* cells, because Pellino1 and Pellino2 can generate the K63-Ub chains required to activate TAK1 heterotrimers in these cells [9]. In contrast, the E3 ligase-inactive TRAF6[L74H] and TRAF6[C70A] mutants failed to restore IL-1 signaling to the TAB2/TAB3/TRAF6 TKO cells (Figure 3B). The expression of TRAF6 and its E3 ligase activity therefore appear to be essential for the activation of the TAB1-TAK1 heterodimer in TAB2/3 DKO IL-1R* cells. Consistent with these findings, the siRNA knockdown of Ubc13 reduced the IL-1β-dependent activation of TAK1 and its substrates in both TAB2/3 DKO and WT IL-1R* cells (Figure 3C). The Ubc13-Uev1 E2-conjugating enzyme forms a productive complex with TRAF6 to direct the specific formation of K63-Ub chains [35].

**Figure 4.**
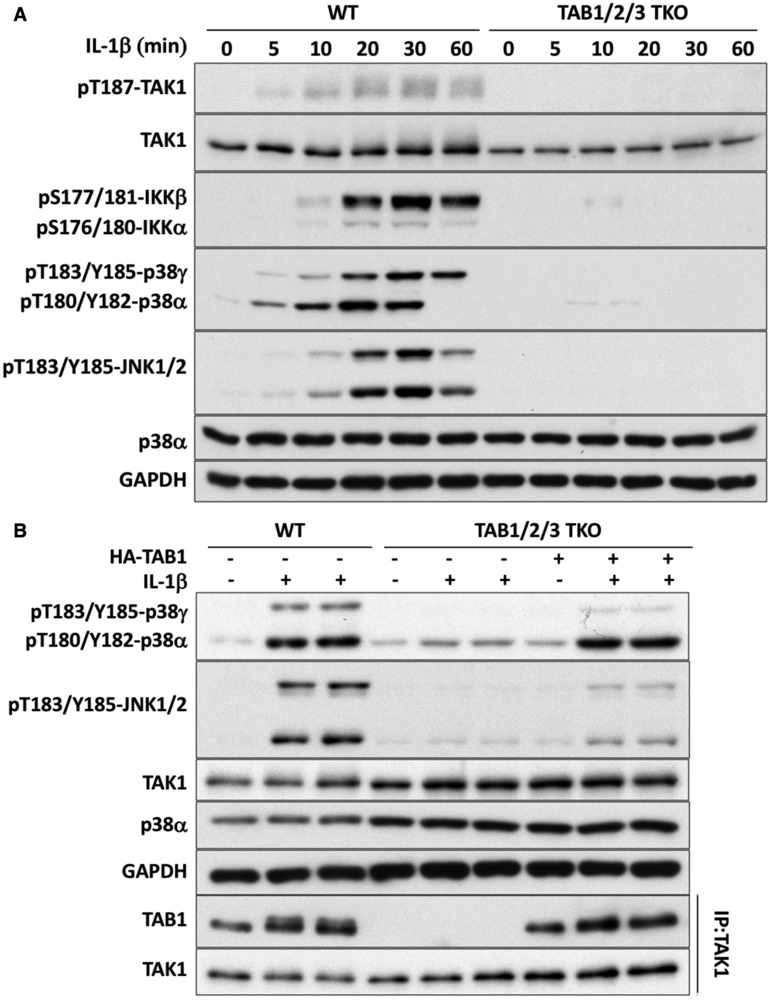
IL-1 signaling is restored to TAB1/2/3 TKO cells by the re-expression of TAB1. (**A**) The experiment was carried out as in Figure 3A, except that TAB1/2/3 TKO cells (clone A4 from Figure 1A) and WT IL-1R* cells were used. (**B**) As in **A**, except that HA-TAB1 was re-expressed in the TAB1/2/3 TKO IL-1R* cells where indicated and the cells stimulated for 10 min with 5 ng/ml IL-1β. Extracts from WT cells (20 µg of protein) and TAB1/2/3 TKO IL-1R* cells (40 µg of protein) were then processed as in **A** (top five panels). In the bottom two panels, TAK1 was immunoprecipitated from the extracts and immunoblotted with anti-TAB1 and anti-TAK1 to confirm that the re-expressed TAB1 had recombined with TAK1.

To assess the importance of TAB1 in the activation of the TAB1–TAK1 heterodimer, we generated IL-1R* cells lacking expression of all three TAB components (Figure 1A, **lanes 5 and 6**). IL-1β did not stimulate the phosphorylation of TAK1 or its substrates in these cells (Figure 4A), but the activation of p38α MAP kinase and the weak activation of JNK1/2 could be restored by the re-expression of TAB1 (Figure 4B). These experiments demonstrate an essential role for TAB1 in permitting IL-1β to activate the TAB1–TAK1 heterodimer.

### XIAP is not required for the activation of the TAB1–TAK1 heterodimer

It has been reported that the E3 ubiquitin ligase XIAP binds to the N-terminus of TAB1, and that WT XIAP but not an XIAP mutant unable to bind to TAB1 induced NF-κB-dependent gene transcription when overexpressed in HEK293T cells [36]. To investigate the potential involvement of XIAP in the IL-1-dependent activation of the TAB1–TAK1 heterodimer, we generated IL-1R* TKO cells lacking any expression of XIAP, TAB2 and TAB3. The IL-1β-dependent activation of TAK1 in XIAP KO cells was slightly enhanced compared with that observed in XIAP-expressing cells (Supplementary Figure S3A). We also eliminated the expression of two other IAP family members (cIAP1 and cIAP2) by incubating the cells with GT12911 [31–33]. These experiments showed that loss of all three IAP family members did not affect the IL-1-dependent activation of the TAB1–TAK1 heterodimer in IL-1R* cells (Supplementary Figure S3B). Taken together, these experiments indicate that XIAP, cIAP1 and cIAP2 are not involved in mediating the IL-1β-dependent activation of the TAB1–TAK1 heterodimer in IL-1R* cells.

### IL-1β does not stimulate the phosphorylation of TAK1 at Ser439

It has been reported that IL-1 stimulates the phosphorylation of Ser439 on TAK1 in IL-1R cells by a protein kinase distinct from TAK1 [37,38] and that the TAK1[S439A] mutant is activated less efficiently by lipopolysaccharide than WT TAK1 when re-expressed in TAK1 KO mouse embryonic fibroblasts [38]. We confirmed using an antibody from Cell Signalling Technology (#9339) that the endogenous TAK1 was phosphorylated at Ser439 in WT IL-1R* cells and TAB2/3 DKO cells, but phosphorylation was not increased by stimulation with IL-1β or suppressed by the TAK1 inhibitor NG25 (Supplementary Figure S4). These experiments indicate that the phosphorylation of Ser439 does not mediate the IL-1β-dependent activation of the TAB1–TAK1 heterodimer in these cells. The IL-1β-dependent phosphorylation of TAK1 at Thr187 and the phosphorylation of IKKα/β, p38α and JNK1/2 were prevented by NG25 as expected [20].

### IL-1β signaling in TAB1 KO IL-1R* cells

**Figure 5.**
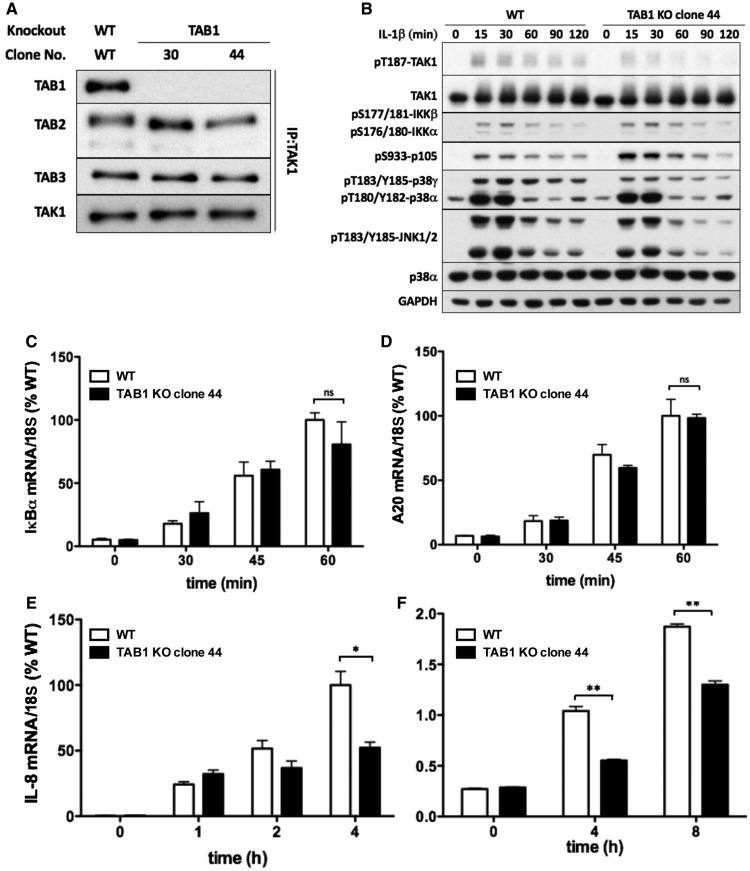
IL-1β signaling in TAB1 KO IL-1R* cells. (**A**) Generation of two clones of TAB1 KO IL-1R* cells. Other details are as in Figure 1A. (**B**) WT IL-1R* cells and TAB1 KO cells (clone 44) were stimulated for up to 2 h with IL-1β . Cell extracts were denatured in SDS, subjected to SDS–PAGE and immunoblotted with the antibodies indicated. (**C**–**E**) As in **B**, except that RNA was extracted from the cells at the times indicated and the formation of IκBα (**C**), A20 (**D**) and IL-8 (**E**) mRNA was measured by qRT-PCR relative to 18S ribosomal mRNA and normalized to the level found in WT cells stimulated with IL-1β. The results are presented as arithmetic mean ± SEM for three independent experiments, each performed in triplicate. (**F**) As in **E**, except that IL-8 secreted into the culture medium was measured by ELISA.

To investigate the regulation of the TAB2–TAK1 and TAB3–TAK1 heterodimers, we next generated TAB1 KO IL-1R* cells (Figure 5A). In these cells, the IL-1β-dependent phosphorylation of TAK1 at Thr187 and the phosphorylation of IKKα/IKKβ and p105/NF-κB1 were similar to WT IL-1R* cells expressing all three TAB subunits (Figure 5B). Consistent with these observations, the IL-1β-dependent transcription of the immediate early genes IκBα (Figure 5C) and A20 (Figure 5D) and the production of IL-8 mRNA (Figure 5E) were similar in TAB1 KO and WT IL-1R* cells for up to 60 min. The IL-1β-dependent phosphorylation of TAK1, JNK1/JNK2, p38α and p38γ MAP kinases was also similar to WT IL-1R* cells for up to 30 min, but modestly reduced after stimulation for an hour or longer (Figure 5B). These observations may explain the small reduction in IL-8 mRNA and IL-8 secretion observed consistently after prolonged stimulation of TAB1 KO cells with IL-1β (Figure 5E,F). Similar results were obtained with a second clone of the TAB1 KO IL-1R* cells that was isolated independently (Supplementary Figure S5).

**Figure 6.**
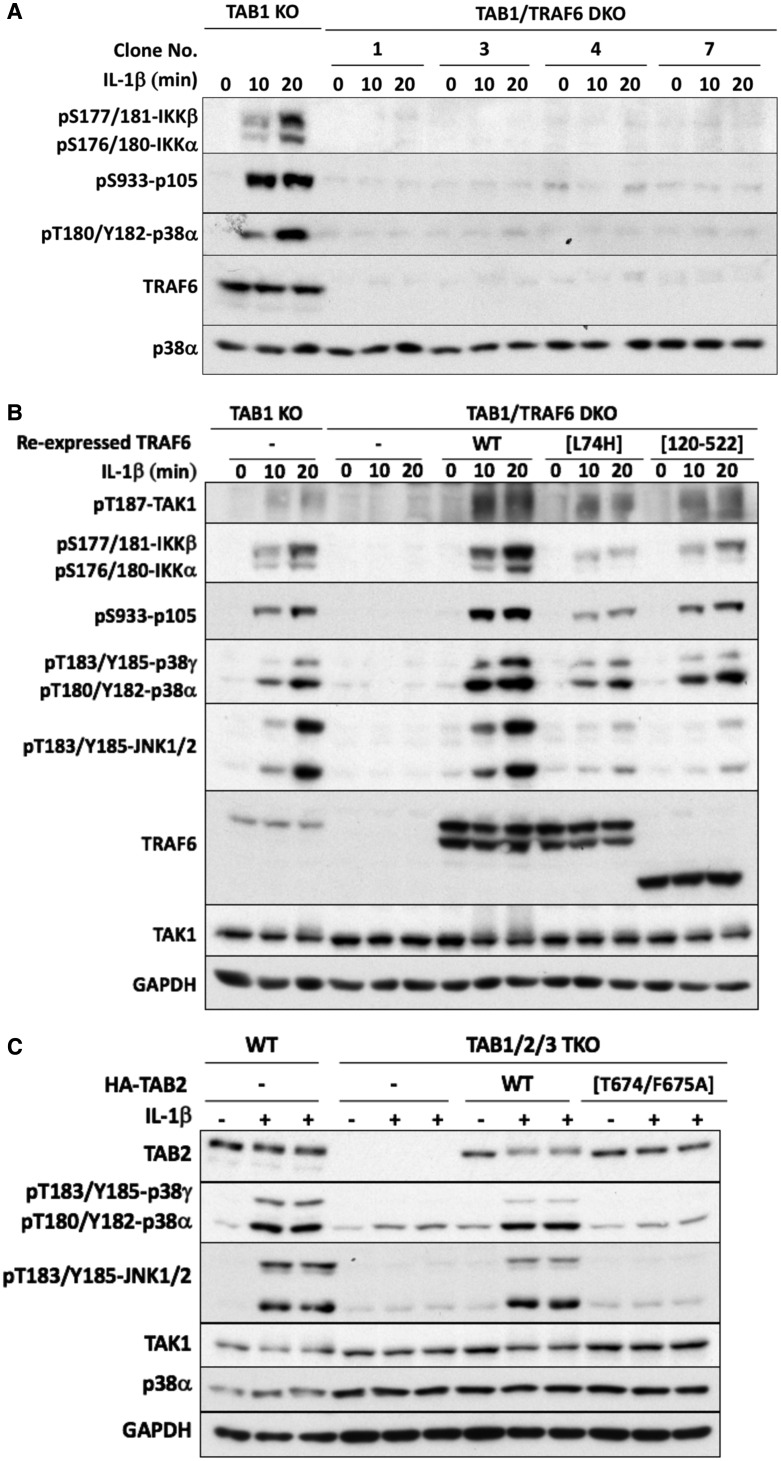
IL-1 signaling in different TAB- and TRAF6-deficient cells. (**A**) Generation of TAB1/TRAF6 DKO cells from TAB1 KO cells (clone 44 from Figure 5A). (**B**) WT TRAF6 and the E3 ligase-inactive TRAF6[L74H] and TRAF6[120-522] mutants were re-expressed in TAB1/TRAF6 DKO cells (clone 1 from **A**). These cells, TAB1 KO cells (clone 44 from Figure 5A) and TAB1/TRAF6 DKO cells not re-transfected with TRAF6, were then stimulated with IL-1β for the times indicated. Other details are as in **A**. (**C**) HA-TAB2 or the K63-Ub-binding-defective HA-TAB2[T674/F675A] mutant were re-expressed in TAB1/2/3 TKO IL-1R* cells (clone A4 from Figure 1A). These cells, TAB1/2/3 TKO cells not re-transfected with HA-TAB2 and WT IL-1R* cells, were stimulated for 10 min with 5 ng/ml IL-1β. Cell extracts (20 µg of protein-WT cells or 40 µg of protein-TAB1/2/3 TKO cells) were subjected to SDS–PAGE and immunoblotted with the antibodies indicated.

To investigate how the TAB2–TAK1 and TAB3–TAK1 complexes were activated, we ablated TRAF6 expression in the TAB1 KO cells to generate TAB1/TRAF6 DKO IL-1R* cells. As expected, no IL-1β-dependent signaling was detectable in these cells (Figure 6A), confirming an essential role for TRAF6 in activating the TAB2–TAK1 and TAB3–TAK1 heterodimers. The re-expression of the E3 ligase-inactive mutant of TRAF6[L74H] mutant, and even a truncation mutant lacking the RING domain of TRAF6 (TRAF6[120-522]), partially restored IL-1β-dependent signaling to TAB1/TRAF6 DKO IL-1R* cells (Figure 6B). Therefore, similar to IL-1R* cells expressing all three TAB components [9], the E3 ligase activity of TRAF6 is not essential for activation of the TAB2–TAK1 and TAB3–TAK1 heterodimers.

To assess the importance of interaction between TAB2 and K63-Ub chains for the activation of the TAB2–TAK1 heterodimer, we re-expressed TAB2 in IL-1R* cells lacking all three TAB components. IL-1β signaling was restored by the re-expression of WT TAB2, but not by a TAB2 mutant, in which Thr673 and Phe674 in the NZF motif were mutated to Ala (Figure 6C). This mutant is unable to interact with K63-Ub chains [29]. Taken together, these experiments indicate that the expression of TAB2 and its interaction with K63-Ub chains is essential for the IL-1β-dependent activation of the TAB2–TAK1 heterodimer in IL-1R* cells devoid of TAB1 and TAB3 expression.

## Discussion

The expression of TRAF6 is required for IL-1 signaling and, until recently, it was thought that the essential role of TRAF6 in this pathway was to generate the K63-Ub chains that interact with TAB2/3 and induce the activation of the TAB1–TAK1–TAB2 and TAB1–TAK1–TAB3 heterotrimers. However, we recently found that the E3 ligase activity of TRAF6 contributes to, but is not essential for IL-1 and Toll-like receptor (TLR) signaling, and that the essential roles of TRAF6 are independent of its E3 ligase activity. This is because, at least in IL-1R* cells, the E3 ligases Pellino1 and Pellino2 operate redundantly with TRAF6 to generate the K63-Ub chains required to initiate IL-1β signaling [9]. Here, we report that the interaction of K63-Ub chains with TAB2 and TAB3 is also dispensable for the acute (<30 min) IL-1β-dependent activation of TAK1 in IL-1R* cells, demonstrating that IL-1β activates the TAB1–TAK1 heterodimer in TAB2/3 DKO cells by another mechanism (Figure 1).

We found that activation of the TAB1–TAK1 heterodimer requires both the expression and the E3 ligase activity of TRAF6 (Figure 3B), but neither TAB1 nor TAK1 possess a known ubiquitin-binding domain. These observations suggest that TRAF6-generated ubiquitin chains activate the TAB1–TAK1 heterodimer indirectly. One possibility is that TRAF6-generated K63-Ub chains might activate an as-yet unidentified protein kinase, which phosphorylates and activates the TAB1–TAK1 heterodimer. However, it is unlikely that such a kinase activates TAK1 by phosphorylating Thr187 directly, because IL-1β does not induce Thr187 phosphorylation in IL-1R* cells expressing the kinase-inactive TAK1[D175A] mutant [9]; instead, this observation suggests that Thr187 is phosphorylated by TAK1 itself. The putative protein kinase might therefore phosphorylate another amino acid residue(s) in TAK1 or TAB1, which induces a conformational change that enables TAK1 to auto-activate. Indeed, it has been reported that TAK1 activity is enhanced by the phosphorylation at Ser439, which is catalyzed by another protein kinase(s) [37,38]. However, we could exclude Ser439 phosphorylation as the mechanism because the phosphorylation of this site was not increased when the TAB2/3 DKO cells were stimulated with IL-1β (Supplementary Figure S4).

A further possibility is that the TAB1–TAK1 heterodimer is intrinsically active, even in cells not stimulated with IL-1β, and that Thr187 phosphorylation is suppressed under basal conditions by the action of one or more serine/threonine-specific protein phosphatases. In support of this contention, the incubation of primary keratinocytes with calyculin A, a potent inhibitor of the *PPP* gene family of serine-/threonine-specific protein phosphatases (PPs), induced the phosphorylation of TAK1 at Thr187 in keratinocytes from WT mice but not in keratinocytes from TAB1 KO mice. PP2A [39] and PP6 [40] were reported to modulate TAK1 phosphorylation and the latter to be associated with the endogenous TAK1 [40]. It is therefore possible that TRAF6-generated K63-Ub chains induce the IL-1β-dependent activation of the TAB1–TAK1 heterodimer by inhibiting a TAK1 phosphatase(s).

Consistent with the findings reported here, Takeuchi and co-workers reported that the phosphorylation of TAK1 and the activation of NF-κB were not impaired in TAB2/TAB3-deficient B cells following stimulation with the TLR9 ligand CpG DNA, with anti-CD40 or with IgM [41]. They also reported that the phosphorylation of JNK was reduced and some late B cell responses were impaired. Based on these observations, they concluded that TAB2 and TAB3 regulate MAP kinases in B cells by a TAK1-independent mechanism. However, their observation that JNK activation was reduced in TAB2/TAB3-deficient cells without an effect on IKK activation is similar to our observations in IL-1-stimulated TAB2/3 DKO IL-1R* cells (Figure 1C). Therefore, an alternative interpretation of their findings is that TAB2 and TAB3 have a specific role in directing the TAB1–TAK1–TAB2 and/or TAB1–TAK1–TAB3 heterotrimers to the MKK family members that activate JNK1 and JNK2. Analogous roles for the regulatory components of other protein kinases and phosphatases are well documented [42]. Similar to TAB2/3, the NF-κB essential modifier (NEMO) component of the canonical IKK complex binds ubiquitin chains, but it also interacts with IκBα, which is critical for the IKKβ-catalyzed phosphorylation of IκBα [43,44]. The loss of some responses in TAB2/3-deficient B cells [41] could also be explained if, as found in the present study, the activation of the TAB1–TAK1 complex is more transient in TAB2/3-deficient B cells than the heterotrimeric TAK1 complexes.

In summary, our results indicate that IL-1β activates TAK1 complexes in IL-1R* cells in two ways, both dependent on the expression of TRAF6: first, by the previously described binding of K63-Ub chains to the NZF domains of TAB2 and TAB3; second, by a ubiquitin-dependent mechanism that is independent of the binding of K63-Ub chains to TAB2 and TAB3 and requires the expression of TAB1. The IL-1β-dependent activation of the TAB1–TAK1 heterodimer is rapid, robust and sufficient for the NF-κB-dependent transcription of immediate early genes (Figure 2). The IL-1β-dependent activation of the TAB2–TAK1 and TAB3–TAK1 heterodimers contributes to the acute activation of TAK1, but is especially important in sustaining signaling for a prolonged period in IL-1R* cells (Figure 5). In contrast with activation of the TAB1–TAK1 heterodimer, the TRAF6 E3 ligase activity is not essential for the activation of TAB2–TAK1 and TAB3–TAK1 complexes (Figure 6B), because the K63-Ub chains that interact with TAB2/3 can also be generated by Pellino1 and Pellino2 [9].

## Abbreviations

Cas9: CRISPR-associated protein 9
cIAP: cellular inhibitor of apoptosis protein
CRISPR: clustered regularly interspaced short palindromic repeats
DKO: double knockout
ERK: extracellular signal regulated kinase
GAPDH: glyceraldehyde 3-phosphatase dehydrogenase
gRNA: guide RNA
HEK293: human embryonic kidney 293 cell
IκBα: inhibitor of kappa B alpha
IKK: IκBα kinase
IL-1: interleukin-1
IL-1R: IL-1 receptor
IRAK: IL-1R-associated kinase
IRF5: interferon regulatory factor 5
JNK: c-Jun N-terminal kinase
K63-Ub: Lys63-linked ubiquitin
MAP: mitogen-activated protein
MAPK: MAP kinase
MEK: mitogen-activated kinase kinase or ERK kinase
MKK: MAP kinase kinase
MRC-PPU: Medical Research Council Protein Phosphorylation and Ubiquitylation Unit
MyD88: Myeloid Differentiation primary response gene 88
NEMO: NF-κB essential modifier
NF-κB: nuclear factor kappa B
NZF: Npl4 zinc finger
RING: Really Interesting New Gene
siRNA: small interfering RNA
TAB: TAK1-binding
TAK1: TGFβ-activated kinase 1
TKO: triple knockout
TLR: Toll-like receptor
Tpl2: tumor progression locus 2
TRAF6: tumor necrosis factor receptor-associated factor 6
Ubc13: ubiquitin conjugating 13
XIAP: X-linked inhibitor of apoptosis protein
