# Supplementary material for: Interleukin-1 and TRAF6-dependent activation of TAK1 in the absence of TAB2 and TAB3
Source: Biochem J. 2017 Jun 26;474(13):2235–48. doi: 10.1042/BCJ20170288 (PMC5632801; doi:10.1042/BCJ20170288)
Supplement: Supplementary Figures [file BCJ-474-2235-s1.pdf]

Supplementary figures

Figure S1. DNA sequences targeted by guide RNAs to make the knock-out cell lines by CRISPR/Cas9 gene-editing technology that were used in the paper.

Figure S2. IL-1β signaling in TAB2/3 double knock-out IL-1R* cells
(**A, B**) Wild type (WT) IL-1R* cells or TAB2/3 double knock-out (DKO) IL-1R* cells (clone 11 from Fig 1A) were stimulated for up to 1 h (**A**) or 2 h (**B**) with 5 ng/ml IL-1β for the times indicated. The cell extracts (20 μg protein) were subjected to SDS/PAGE and after transfer to PVDF membranes, were immunoblotted with the antibodies indicated. (**C**) IL-8 mRNA was measured by qRTPCR relative to 18S ribosomal mRNA and normalized to the level found in wild-type cells stimulated with IL-1β. The results are presented as arithmetic mean ± SEM for three independent experiments, each performed in triplicate. **(D)** As in C, except that IL-8 secreted into the culture medium was measured by ELISA.

Figure S3. XIAP and cIAP1/2 are not required to activate the TAB1-TAK1 heterodimer in TAB2/3 double KO IL-1R* cells.
(**A, B**) TAB2/3 double knock-out (DKO) IL-1R*cells (clone 4 from Fig 1A) and TAB2/TAB3/XIAP triple knock-out (TKO) cells were incubated for 1 h without **(A)** or with **(B)** GT12911 (100 nM), and then stimulated with (+) or without (-) 5 ng/ml IL-1β for the times indicated. Cell extract protein (20 μg) was subjected to SDS/PAGE, followed by transfer to PVDF membranes and immunoblotting with the antibodies indicated.

Figure S4. IL-1β stimulation does not enhance TAK1 phosphorylation at Ser439.
Wild type IL-1R* cells or TAB2/3 double knock-out (DKO) IL-1R*cells (clone 4 from Fig 1A) were incubated for 1 h with (+) or without (-) the indicated concentrations of the TAK1 inhibitor NG25, then stimulated for 10 min with IL-1β. Cell extract (20 μg protein) was subjected to SDS-PAGE followed by immunoblotting with the antibodies indicated.

Figure S5. IL-1β signaling and gene transcription in TAB1 KO IL-1R* cells.
(**A**)Wild type (WT) IL-1R* cells or TAB1 KO IL-1R* cells (clone 30 from Fig 5A) were stimulated with 5 ng/ml IL-1β for the times indicated, then subjected to SDS/PAGE and immunoblotting with the antibodies indicated. **(B)** The experimentwas performed as in A except that RNA was extracted from the cells at the times indicated and the formation of IL-8 mRNA was measured by qRTPCR relative to 18S ribosomal mRNA and normalized to the level found in wild-type cells stimulated with IL-1β. The results are presented as arithmetic mean ± SEM for three independent experiments, each performed in triplicate. **(C)** As in A except that the IL-8 secreted into the culture medium was quantified by ELISA.

Figure S1

Gene	Exon	DNA sequence	DU Number
TAB1	1	GGAGCTTGCTGCAGAGTGTGAGG	48411
TAB1	2	GCCACCCGCCAGAGGACAGC (sense)	52383
TAB1	2	GATCAGCAGAGTAGCTGCGGT (antisense)	52393
TAB2	4	GCTCTGCAACCTCTCCTCGAG(sense)	52384
TAB2	4	GATTGCCTATTGCTCGACTTT(antisense)	52394
TAB3	7	GTTGGTATCACTCGAGGAGATGG	48354
TRAF6	2	GAAGCAGTGCAAACGCCATG (sense)	52382
TRAF6	2	GTCGTAATGCCATCAAGCAGA(antisense)	52392
XIAP	2	GCAGACTATCTTTTGAGAACT (sense)	52462
XIAP	2	GTGTCTCAGATGGCCTGTCTA (antisense)	52474

Figure S2

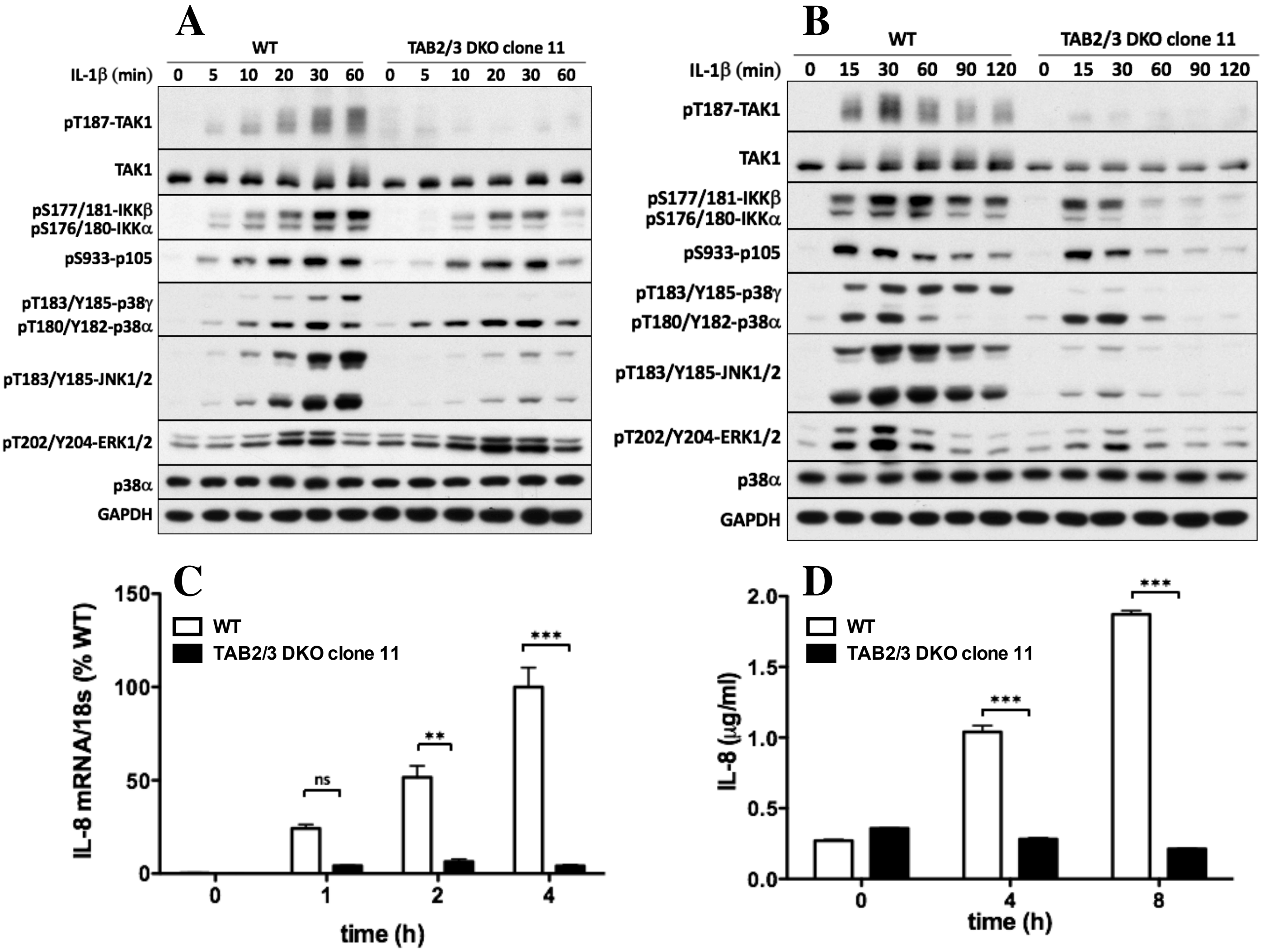
A
WT
TAB2/3 DKO clone 11
IL-1β (min) 0 5 10 20 30 60 0 5 10 20 30 60
pT187-TAK1
TAK1
pS177/181-IKKβ
pS176/180-IKKα
pS933-p105
pT183/Y185-p38γ
pT180/Y182-p38α
pT183/Y185-JNK1/2
pT202/Y204-ERK1/2
p38α
GAPDH
B
WT
TAB2/3 DKO clone 11
IL-1β (min) 0 15 30 60 90 120 0 15 30 60 90 120
pT187-TAK1
TAK1
pS177/181-IKKβ
pS176/180-IKKα
pS933-p105
pT183/Y185-p38γ
pT180/Y182-p38α
pT183/Y185-JNK1/2
pT202/Y204-ERK1/2
p38α
GAPDH
C
IL-8 mRNA/18s (% WT)
WT
TAB2/3 DKO clone 11
ns
**
***
time (h)
D
IL-8 (μg/ml)
WT
TAB2/3 DKO clone 11
***
***
time (h)

Figure S3

A

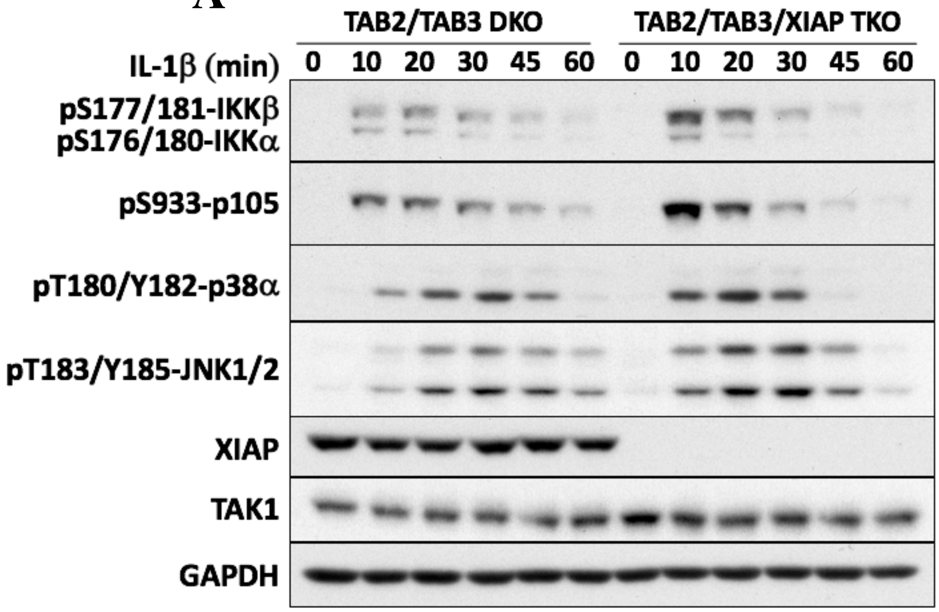
TAB2/TAB3 DKO
TAB2/TAB3/XIAP TKO
IL-1β (min)
0 10 20 30 45 60 0 10 20 30 45 60
pS177/181-IKKβ
pS176/180-IKKα
pS933-p105
pT180/Y182-p38α
pT183/Y185-JNK1/2
XIAP
TAK1
GAPDH

B

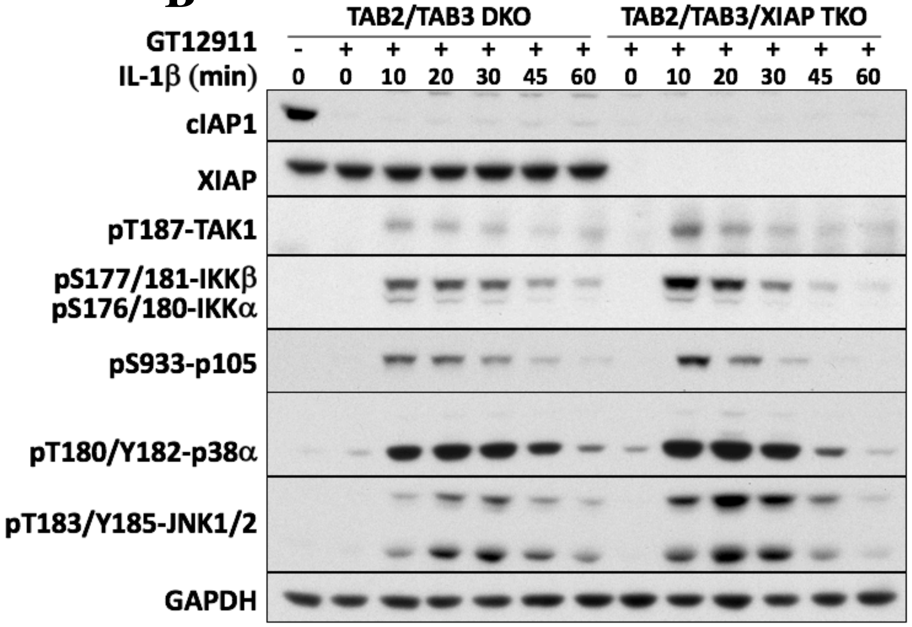
TAB2/TAB3 DKO
TAB2/TAB3/XIAP TKO
GT12911
- + + + + + + + + + + + +
IL-1β (min)
0 0 10 20 30 45 60 0 10 20 30 45 60
cIAP1
XIAP
pT187-TAK1
pS177/181-IKKβ
pS176/180-IKKα
pS933-p105
pT180/Y182-p38α
pT183/Y185-JNK1/2
GAPDH

Figure S4

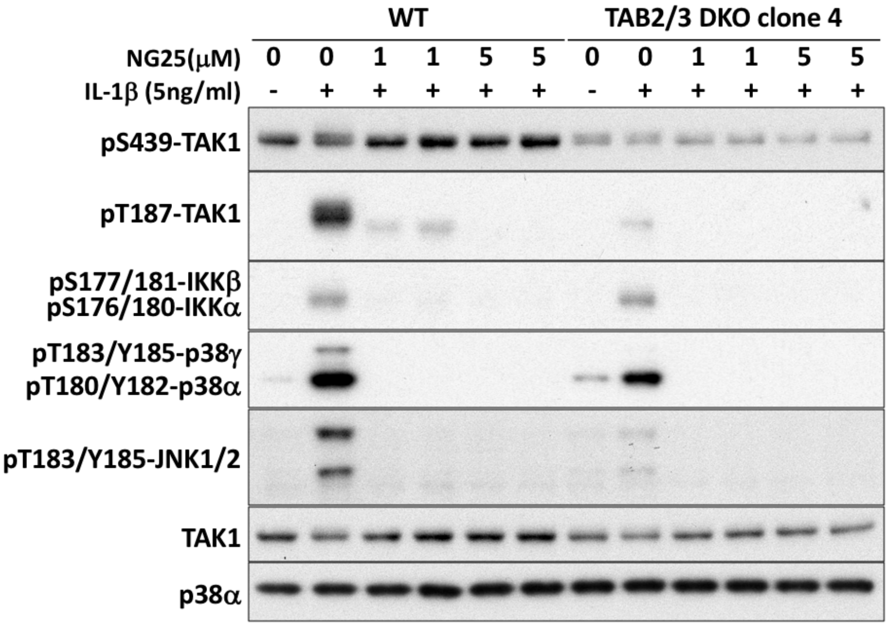
WT
TAB2/3 DKO clone 4
NG25(μM) 0 0 1 1 5 5 0 0 1 1 5 5
IL-1β (5ng/ml) - + + + + + - + + + + +
pS439-TAK1
pT187-TAK1
pS177/181-IKKβ
pS176/180-IKKα
pT183/Y185-p38γ
pT180/Y182-p38α
pT183/Y185-JNK1/2
TAK1
p38α

Figure S5

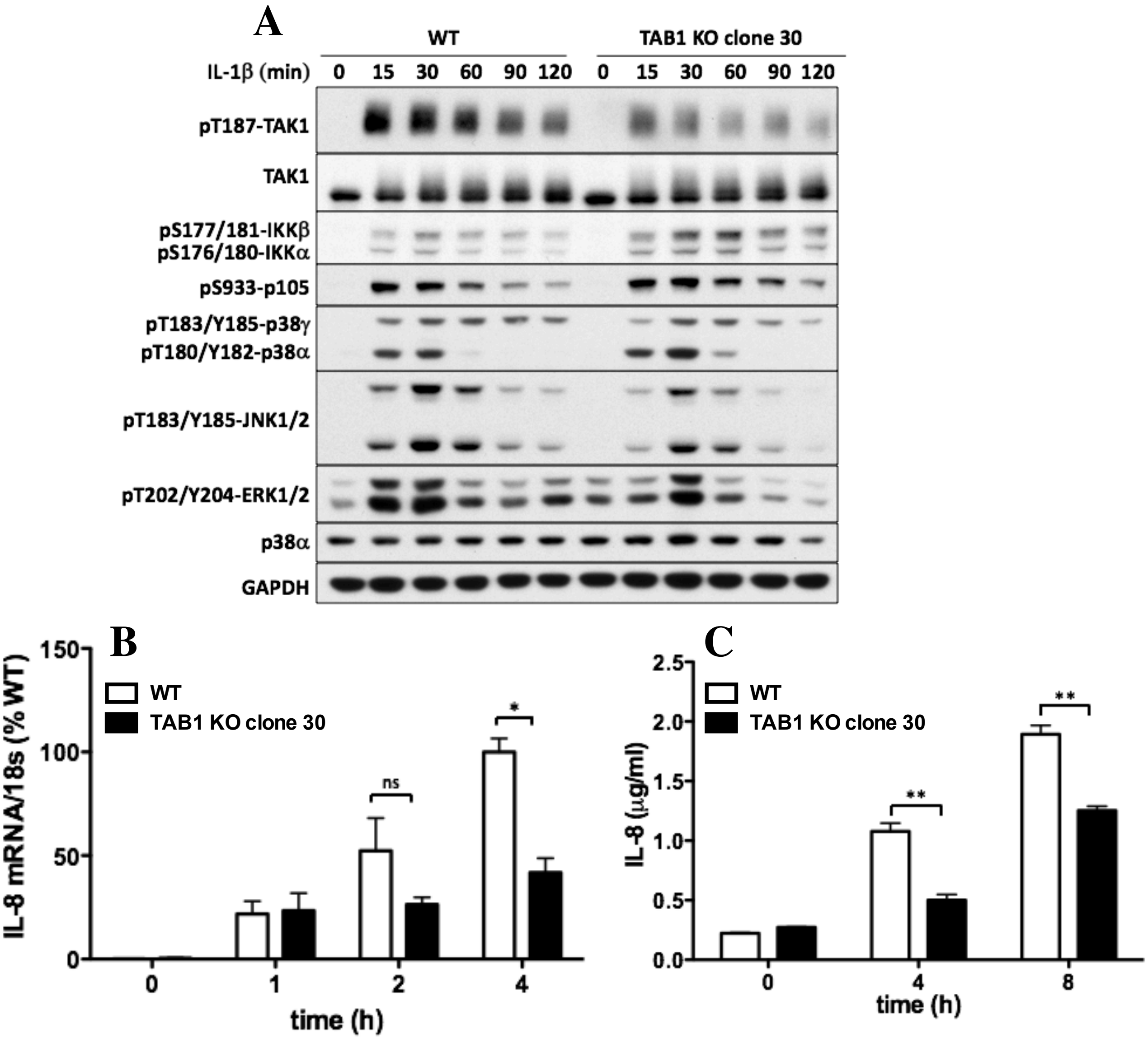

A
WT
TAB1 KO clone 30
IL-1β (min) 0 15 30 60 90 120 0 15 30 60 90 120
pT187-TAK1
TAK1
pS177/181-IKKβ
pS176/180-IKKα
pS933-p105
pT183/Y185-p38γ
pT180/Y182-p38α
pT183/Y185-JNK1/2
pT202/Y204-ERK1/2
p38α
GAPDH
B
WT
TAB1 KO clone 30
IL-8 mRNA/18s (% WT)
150
100
50
0
ns
*
0
1
2
4
time (h)
C
WT
TAB1 KO clone 30
IL-8 (µg/ml)
2.5
2.0
1.5
1.0
0.5
0.0
**
**
0
4
8
time (h)
